# Acute neuromuscular and kinematic responses to a single Feldenkrais awareness through movement session

**DOI:** 10.3389/fnhum.2026.1924684

**Published:** 2026-10-07

**Authors:** Eisa Alokla, Roger Russell, André Seyfarth, Maziar Ahmad Sharbafi

**Affiliations:** 1 Lauflabor, Locomotion Laboratory, Institute of Sports Science and Center for Cognitive Science, Technische Universität Darmstadt, Darmstadt, Germany; 2 Feldenkrais Center Heidelberg, Heidelberg, Germany; 3 Control and Cyber-Physical Systems Laboratory, Department of Electrical Engineering and Information Technology, Technische Universität Darmstadt, Darmstadt, Hessen, Germany

**Keywords:** awareness through movement, exploratory motor learning, Feldenkrais method, human movement analysis, intersegmental coordination, movement quality, movement smoothness, neuromuscular adaptation

## Abstract

**Introduction:**

The Feldenkrais Method aims to improve functional movement through exploratory coordination rather than performance-driven execution. While clinical studies have reported benefits in mobility and balance, the underlying neuromechanical mechanisms remain insufficiently characterized.

**Methods:**

This study examined acute changes in voluntary range of motion (ROM), muscle activation, movement smoothness, and ventilatory—movement efficiency following a single Awareness Through Movement session in healthy adults (intervention: *n* = 44; control: *n* = 12) using three-dimensional motion capture, surface electromyography, and capnography.

**Results:**

The intervention group showed significant within-group increases in upper-body ROM, selective increases in muscle activation in trunk and neck muscles, a significant increase in forearm angle, and a reduction in VMEI following the session. None of these changes were observed in the control group. Time × Group analyses confirmed intervention-specific effects for shoulder ROM, forearm angle, and right sternocleidomastoid activation.

**Discussion:**

These findings suggest that short-term Feldenkrais-based exploratory practice produces coordinated changes in movement organization and neuromuscular activation that are not explained by repeated movement practice alone, with ROM changes consistent with altered intersegmental coordination rather than passive mechanical effects.

## 1 Introduction

Functional movement can improve not only through changes in strength or flexibility, but also through changes in how coordination is organized across body segments. This perspective is particularly relevant in movement-based therapeutic approaches, where improvements in mobility and movement quality may emerge from altered movement strategies rather than from increased physical capacity alone. Within this context, the Feldenkrais Method, especially its “Awareness Through Movement” (ATM) lessons, has been used as an exploratory approach to improve functional movement through attention, variation, and self-regulated coordination rather than performance-driven instruction (Buchanan and Ulrich, 2001; Russell, 2020). In ATM, participants are encouraged to move with reduced effort, attend to sensory feedback, and explore alternative movement solutions rather than reproduce prescribed patterns (Stephens and Hillier, 2020; Lyttle, 1997). From a motor learning perspective, such practice is commonly understood as influencing movement organization and neuromuscular coordination rather than simply increasing range through mechanical stretching (Newell, 1990; Mattes, 2016). This view is consistent with broader concepts of movement quality, in which functional improvement depends not only on how much motion is achieved, but also on how that motion is distributed and coordinated across multiple segments and degrees of freedom (Wulf, 2013; Bernstein, 1967). Empirical studies have reported beneficial effects of Feldenkrais-based interventions on mobility, balance, pain, and functional performance across different populations (Ullmann et al., 2010). Ullmann et al. (2010) found improvements in balance and mobility in community-dwelling older adults following a 5-week ATM program. A systematic review in musculoskeletal rehabilitation reported supportive evidence in individuals with neck and low back disorders, although methodological heterogeneity limited firm biomechanical conclusions (Henry et al., 2016). A subsequent meta-analysis confirmed improvements in mobility, balance, pain, and functional performance, while also noting that much of the literature relies on clinical outcomes rather than objective movement analysis (Berland et al., 2022). Thus, although the intervention literature is promising, the mechanisms through which Feldenkrais-based practice may alter movement remain insufficiently characterized.

This gap is important because therapeutic movement approaches are often evaluated through clinical scales or self-reported function, while comparatively little is known about associated changes in movement coordination and neuromuscular organization. Previous review-based work has suggested improvements in motor function and range of motion following Feldenkrais-based interventions, but these conclusions have largely relied on aggregated outcomes rather than direct experimental analysis of human movement and muscle activation (Hillier and Worley, 2015). Exploratory use of motion-tracking technologies has been reported (Beacon, 2015), yet laboratory-based studies integrating three-dimensional kinematics with surface electromyography remain scarce. It is therefore still unclear to what extent reported improvements reflect altered intersegmental coordination, modified muscle recruitment, or non-specific effects of repeated movement practice.

From a theoretical standpoint, this question can be framed within motor learning and motor control perspectives in which habitual movement patterns remain adaptable through experience. In this sense, exploratory movement practice may facilitate redistribution of motion across body segments and more flexible use of available degrees of freedom, consistent with Bernstein's account of motor coordination during skill acquisition (Newell, 1990; Bernstein, 1967). Such ideas are frequently invoked in discussions of Feldenkrais practice and are relevant to rehabilitation because they suggest that functional changes in voluntary range of motion (ROM) may arise from altered coordination strategies rather than from passive tissue change alone.

Beyond ROM and muscle activation, the quality of voluntary movement trajectories may also be influenced by exploratory practice. Minimum-jerk formulations, which describe smooth voluntary movements as minimizing the rate of change of acceleration, offer a motor-control framework for quantifying trajectory quality independently of movement speed and amplitude (Flash and Hogan, 1985). Feldenkrais principles have also been discussed in relation to kinaesthetic awareness and adaptive sensory engagement with movement (Igweonu, 2010; Feltham and Loke, 2014). These perspectives suggest that ATM practice may influence not only how much motion is produced, but also how efficiently and smoothly that motion is coordinated. Despite this theoretical basis, objective laboratory evidence directly linking ATM practice to measurable kinematic, neuromuscular, and ventilatory changes under controlled conditions remains scarce.

The present study examines acute changes in voluntary ROM, muscle activation, movement smoothness, and ventilatory–movement efficiency following a single ATM session in healthy adults, using three-dimensional kinematic analysis, surface electromyography, and capnography. The experimental task is illustrated in Figure 1. Within a controlled pre–post design with an active control group, we tested whether observed changes are specific to the Feldenkrais-based exploratory practice rather than the result of repeated movement execution. We hypothesized that the ATM intervention would produce intersegmental coordination changes reflected in ROM, forearm angle, muscle activation, and movement efficiency that would not be observed in the control group.

**Figure 1:**
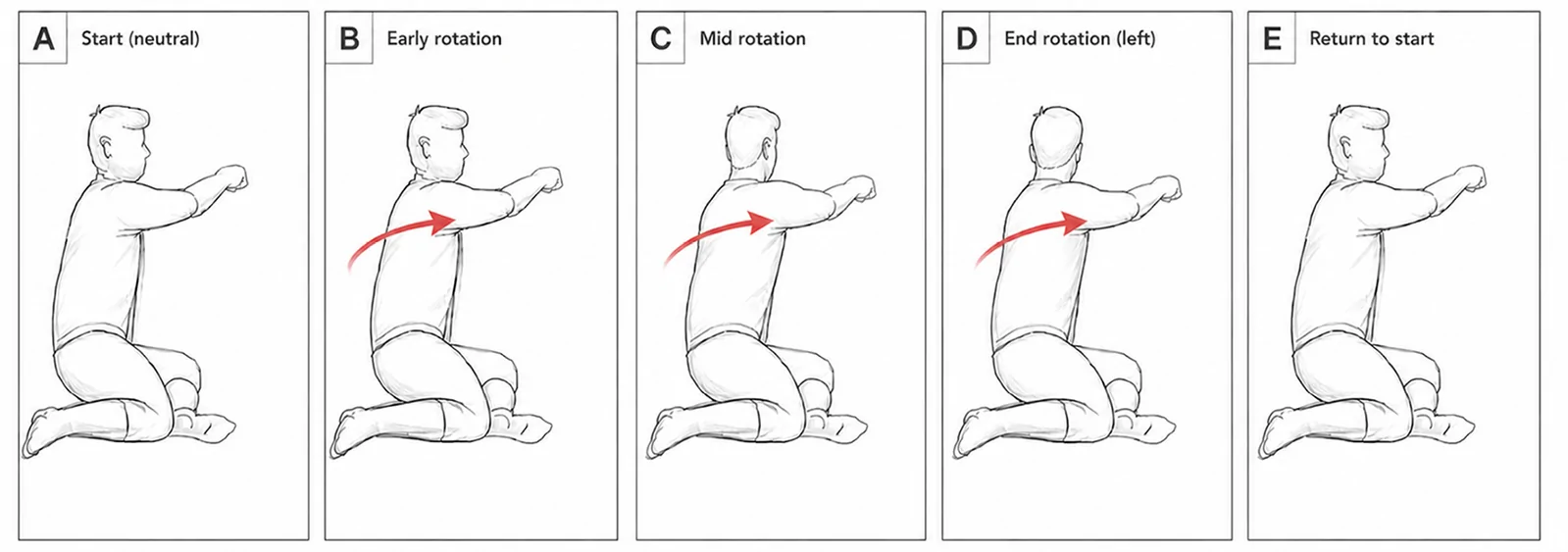
Standardized seated trunk-rotation task during the Awareness Through Movement (ATM) intervention. **(A–E)** Temporal sequence of the movement: **(A)** initial upright sitting posture (reference position), **(B, C)** progressive axial rotation of the trunk toward the left, involving coordinated motion of the trunk, head, and upper limb, **(D)** end position of rotation, and **(E)** return to the initial posture. Participants maintained a stable seated posture throughout the task to ensure consistent pelvic support and controlled upper-body motion.

## 2 Methods

### 2.1 Participants and experimental design

Fifty-eight healthy adults were initially recruited for this controlled pre–post study. Of these, 46 were assigned to the intervention group and 12 to the control group. The final analyzed sample comprised 56 participants (44 intervention, 12 control). Participant demographic and anthropometric characteristics of the analyzed sample are summarized in Table 1. Inclusion criteria were no prior experience with the Feldenkrais Method^®^, no musculoskeletal injury within the preceding 6 months, and the ability to complete the experimental tasks without excessive fatigue. Participants with diagnosed musculoskeletal or neurological disorders were excluded. Forty-six participants were assigned to the intervention group and twelve to the control group. Both groups were recruited through the same general procedure: participants were invited to take part in a movement study and provided informed consent without prior knowledge of the specific experimental protocol. The protocol assigned to each participant was explained only after consent was obtained, and group assignment reflected session attendance. Participants were naive to condition-specific expectations at the time of enrolment. The group size difference reflects the measurement design: all 44 analyzed intervention participants completed the kinematic and EMG measurements, while a subsample of 12 of these participants additionally completed the capnography measurements. All 12 control participants completed the corresponding measurements, including capnography (Voelcker-Rehage et al., 2011). The control group was therefore recruited to provide a comparison for the capnography subgroup rather than to match the full intervention sample. The sample size was determined by the availability of participants enrolled in ATM sessions during the study period; no formal power calculation was performed. A post-hoc sensitivity analysis based on the final analyzed sample (intervention: *n* = 44; control: *n* = 12) is reported in Section 4.6.

**Table 1:** Baseline demographic and anthropometric characteristics of the analyzed intervention and control groups (*N* = 56), compared using Welch's t-test (continuous variables) and a chi-square test (sex distribution).

Characteristic	Intervention (*n* = 44)	Control (*n* = 12)	*p*
Age (years)	22.57 ± 3.98	21.92 ± 2.02	0.44
Height (cm)	174.48 ± 8.82	178.58 ± 11.14	0.26
Body mass (kg)	68.80 ± 12.72	69.75 ± 13.40	0.83
Female, n (%)	21 (47.7%)	5 (41.7%)	0.72
Male, n (%)	23 (52.3%)	7 (58.3%)	—

All participants provided written informed consent before participation. The study was conducted in accordance with the Declaration of Helsinki and approved by the Ethics Committee of Technische Universität Darmstadt (approval number: EK 94/2024).

The intervention group attended a single laboratory session of approximately 2 h, including preparation and instrumentation, with about 45 to 60 min dedicated to a structured Feldenkrais Awareness Through Movement (ATM) session consisting of 15 progressive steps. Participants were instructed to explore the movements at a comfortable pace without explicit performance goals or ROM targets (Stephens and Hillier, 2020; Lyttle, 1997). Kinematic and electromyographic (EMG) measurements were collected for all sessions.

The control group underwent the same pre–post measurements under comparable laboratory conditions but did not receive the ATM intervention. Instead, they performed repeated voluntary trunk rotations corresponding to Step 1 of the movement protocol, matched in total duration and repetition count to the intervention group (15 identical movement blocks of approximately 10 repetitions each, lasting 1–2 min per block). The control session was supervised by the same certified Feldenkrais practitioner and included the same short rest intervals between movement blocks as the intervention session. No exploratory instructions, attentional cues, or segmental differentiation tasks were provided. This design was chosen to examine whether the observed changes extend beyond movement repetition alone, thereby providing a basis for interpreting any group differences in relation to the specific content of the ATM intervention rather than movement volume or practitioner presence. The control group was sized to test whether repeated movement practice alone produces the observed changes, not to perform a between-group interaction test; This design was used to examine acute changes in joint range of motion (ROM) and neuromuscular activation following the Feldenkrais-based session, extending earlier kinematic findings (Alokla et al., 2025).

### 2.2 Feldenkrais-based intervention protocol

The intervention consisted of a structured ATM lesson focused on seated trunk rotation and upper-body coordination. The session was supervised by a certified Feldenkrais practitioner and followed core principles of the Feldenkrais Method^®^, including exploratory movement, self-regulation of effort, and attention to internal sensory feedback rather than external correction (Stephens and Hillier, 2020; Lyttle, 1997).

Participants were instructed to move slowly and comfortably, without stretching or force, and to stop at the perceived end of movement. No explicit performance goals or ROM targets were given.

The lesson emphasized progressive differentiation and reorganization of rotational coordination among the pelvis (P), shoulders (S), head (H), and eyes (E), aiming to refine intersegmental control of spinal degrees of freedom. Short rest periods were incorporated to facilitate perceptual integration and minimize unnecessary muscular effort. Each exercise lasted approximately 1–2 min or 10 repetitions, following a stepwise exploration of combined P/S/H/E movements. Movements gradually introduced differentiation of segments, allowing participants to notice subtle changes in their movement and coordination. Optional reference exercises included repeated rotations to the opposite side to allow comparison of range of motion between sides.

Selected exercises and their corresponding initial positions and movement execution are illustrated in Figure 2, providing a schematic overview of the progressive coordination strategy used during the ATM session.

**Figure 2:**
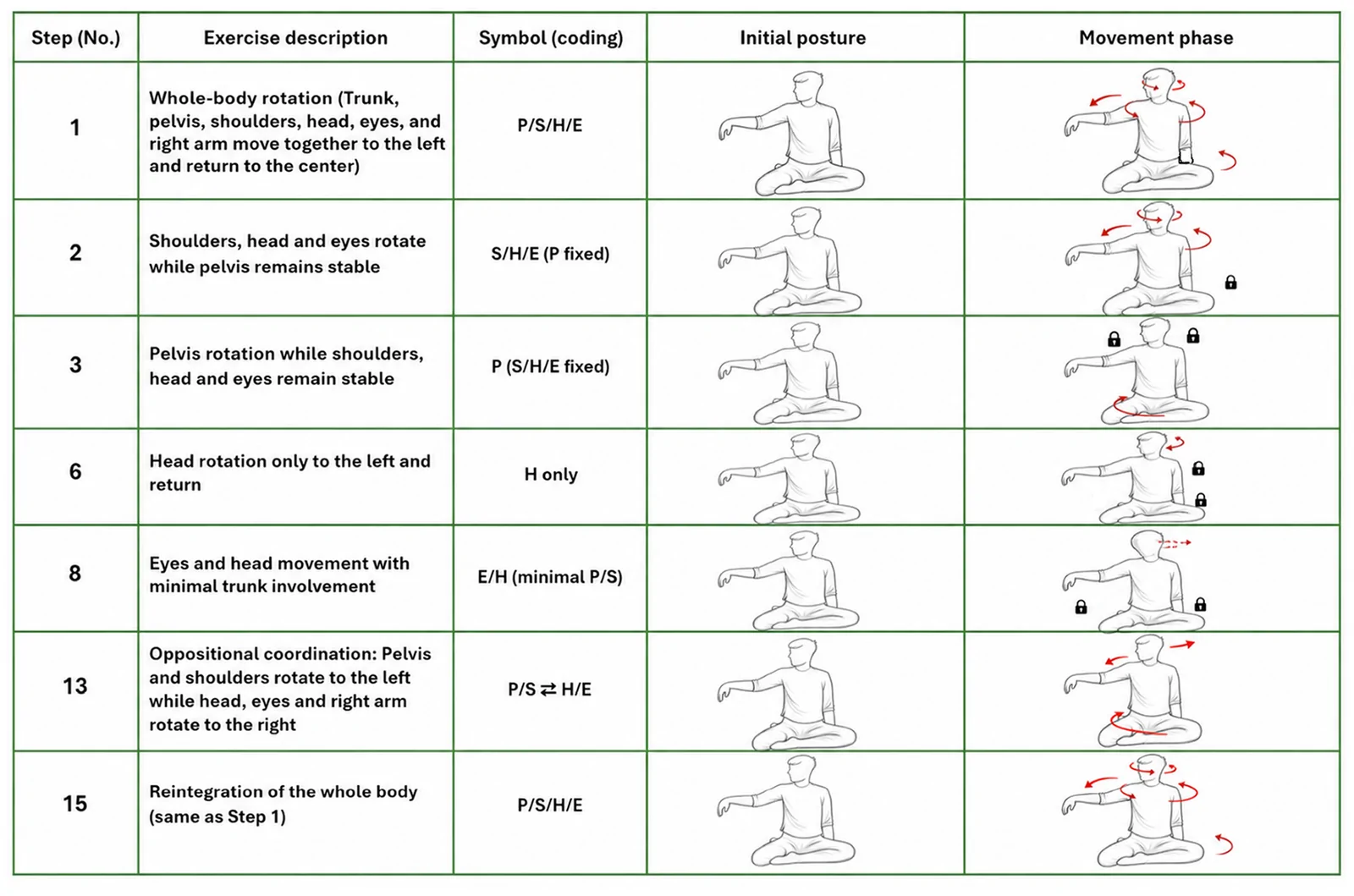
Overview of selected exercises from the structured ATM session, illustrating the initial body position and movement execution for representative steps. P, pelvis; S, shoulders; H, head; E, eyes.

### 2.3 Kinematic and electromyographic measurements

Three-dimensional kinematic data were recorded with a multi-camera optoelectronic motion capture system (Qualisys AB, Gothenburg, Sweden), using the same general setup as in previous work (Alokla et al., 2025). Marker trajectories were sampled at 460 Hz during a standardized seated trunk-rotation task performed before, during, and after the intervention.

A total of 24 reflective markers were placed on anatomical landmarks of the head, shoulders, elbows, wrists, chest, spine, and sacrum (Figure 3). Marker placement included three markers on the anterior head, two on each shoulder, elbow, and wrist, two on the chest, multiple markers along the spine (C7, T2, T4, T5, T7, T11, L1, L4), and three on the sacrum. This configuration was used to quantify upper-body and trunk motion during axial rotation.

**Figure 3:**
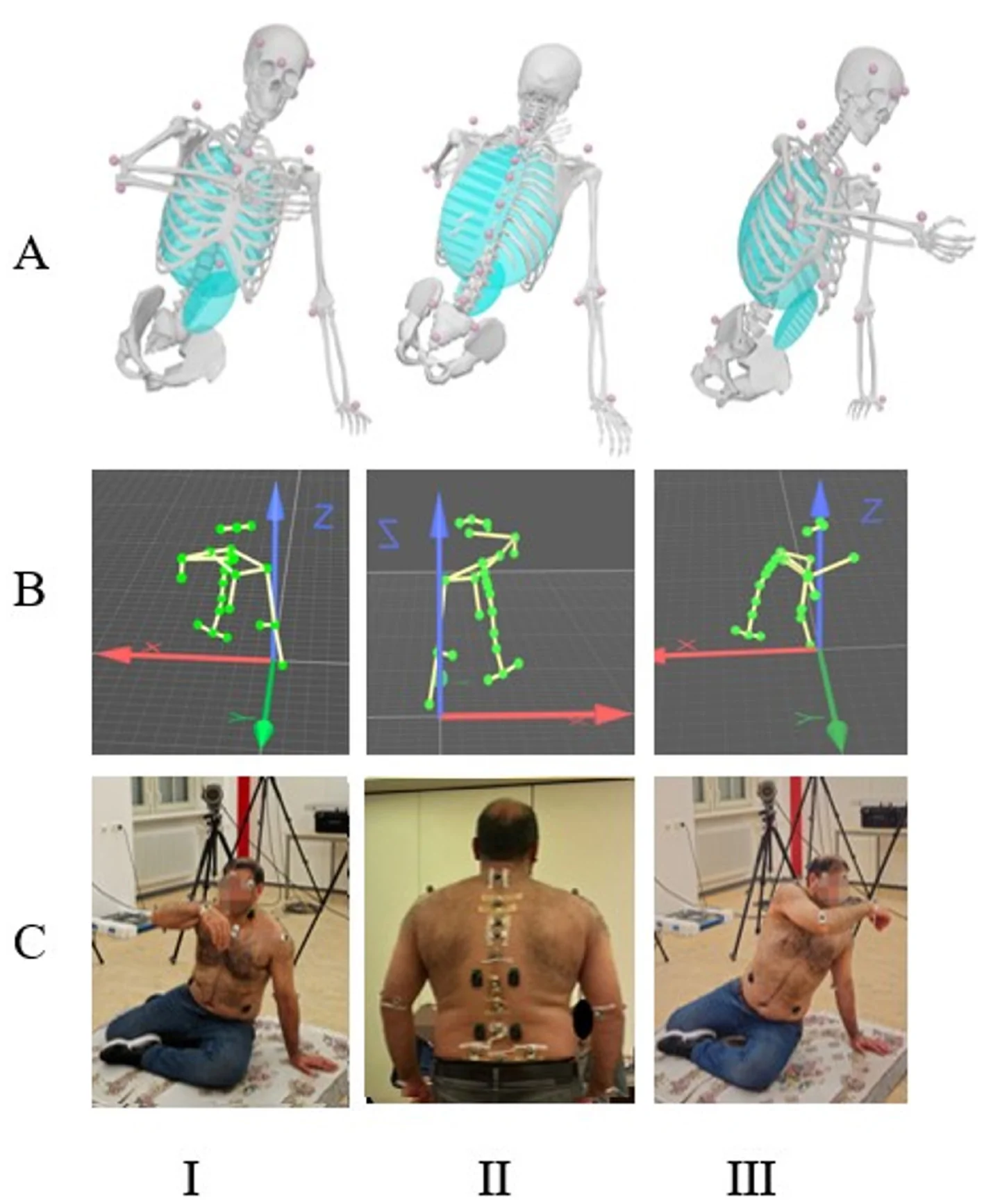
Experimental setup and the movement model. **(A)** conceptual biomechanical marker configuration, **(B)** three-dimensional kinematic reconstruction in Qualisys Track Manager (QTM; Qualisys AB, Gothenburg, Sweden), and **(C)** experimental setup during the seated Feldenkrais trunk-rotation task indicating the placement of reflective markers (white) and surface EMG electrodes (black). Columns I–III illustrate the anterior view (I), posterior view (II), and anterior view during axial trunk rotation (III).

#### 2.3.1 Computation of angular displacement and range of motion

Angular displacement was derived from the three-dimensional marker trajectories using a vector-based approach. For each movement cycle, the instantaneous angle θ between a target vector **v**_*t*_ and a fixed reference vector **v**_*r*_ was computed as


$$\begin{array}{l} \theta =\cos^{-1}\left(\frac{{\text{v}}_{t}\cdot {\text{v}}_{r}}{\left||{\text{v}}_{t}||||{\text{v}}_{r}|\right|}\right) \end{array}$$ (1)

where **v**_*t*_ = **x**_target_(*t*)−**x**_PSIS_(*t*) is the position vector from the sacral reference marker (PSIS-L) to the target marker, and **v**_*r*_ = **x**_target_(*t*_0_)−**x**_PSIS_(*t*_0_) is the corresponding reference vector at the initial time frame. The computed angle therefore reflects the change in target-marker orientation relative to its initial pelvis-referenced configuration.

The movement within each exercise step consisted of continuous oscillatory trunk rotation producing a periodic angular displacement signal with clearly defined peaks and valleys corresponding to the rotational extremes of each repetition. ROM was computed as the difference between the mean peak and mean valley angular values across repetitions within each step. Peak and valley detection was implemented using MATLAB's findpeaks function with a minimum prominence threshold to exclude small signal fluctuations from being detected as genuine movement extrema. This procedure is consistent with standard practice in three-dimensional human motion analysis (Winter, 2009; Wu et al., 2005).


$$\begin{array}{l} ROM={\bar{\theta }}_{\text{peaks}}-{\bar{\theta }}_{\text{valleys}}, \end{array}$$ (2)

where ${\bar{\theta }}_{\text{peaks}}$ and ${\bar{\theta }}_{\text{valleys}}$ denote the mean values of detected angular peaks and valleys, respectively, identified using prominence thresholds selected empirically during visual inspection of individual angular-displacement trajectories, with values ranging from approximately 4° to 6° for peaks and 3° to 5° for valleys across markers and participants. This procedure follows standard principles in three-dimensional human motion analysis (Winter, 2009; Wu et al., 2005).

#### 2.3.2 Computation of forearm angle

To further characterize segmental coordination of the upper limb during the rotation task, a forearm angle was computed based on the relative orientation between the upper arm and forearm segments. The upper arm vector was defined from the shoulder marker to the elbow marker, whereas the forearm vector was defined from the elbow marker to the wrist marker.

The instantaneous forearm angle, denoted as ψ, was calculated as the angle between these two segment vectors using the same vector-based formulation described in Equation 1. Accordingly, ψ represents the local inter-segmental configuration at the elbow joint.

Unlike the angular displacement used for range of motion estimation, which reflects changes in global orientation relative to a fixed reference, the forearm angle provides a joint-specific measure of segmental contribution during movement. This distinction allows separation between global movement amplitude and local coordination strategies of the upper limb.

### 2.4 Electromyographic (EMG) signal processing

Surface EMG was recorded from muscles relevant to trunk and postural control: bilateral sternocleidomastoid (Sterno-R, Sterno-L), abdominal obliques (Obli-R, Obli-L), erector spinae (Erec-Low-R, Erec-Low-L, Erec-High-R, Erec-High-L), and right soleus (Sol-R) (Figure 4). The soleus was included because of its contribution to postural control during voluntary movement tasks (Winter, 2009).

**Figure 4:**
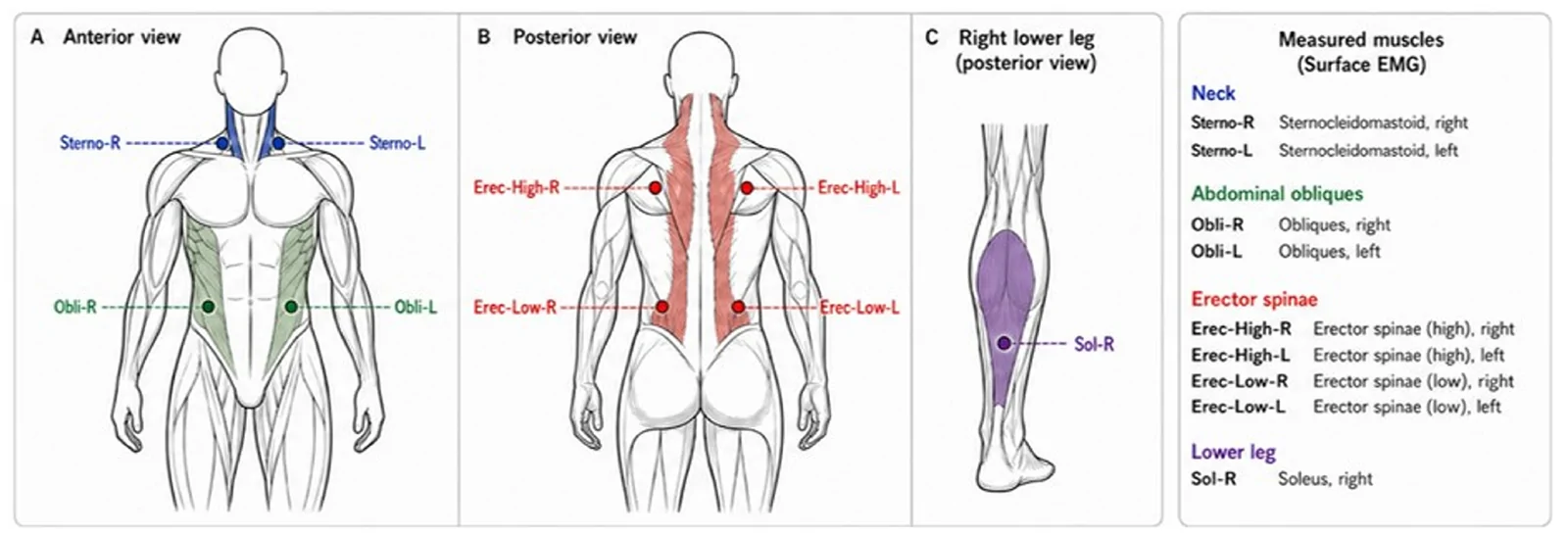
Electrode placement for surface EMG recordings. **(A)** Anterior view showing bilateral sternocleidomastoid (Sterno-R, Sterno-L) and abdominal oblique (Obli-R, Obli-L) muscles. **(B)** Posterior view showing bilateral erector spinae muscles at high (Erec-High-R, Erec-High-L) and low (Erec-Low-R, Erec-Low-L) thoracolumbar levels. Four electrodes were placed bilaterally along the spine to target the erector spinae. **(C)** Posterior view of the right lower leg showing the soleus (Sol-R) muscle.

Signals were acquired using a wireless multi-channel system (Delsys Trigno^®^, USA) following SENIAM recommendations for electrode placement (Hermens et al., 2000). EMG data were sampled at 2000 Hz. Raw signals were band-pass filtered between 10 and 450 Hz with a zero-lag fourth-order Butterworth filter, followed by a 50 Hz notch filter (6 Hz bandwidth). Signals were then full-wave rectified and low-pass filtered at 8 Hz to obtain the linear envelope (Hermens et al., 2000; De Luca, 1997; Merletti and Parker, 2004; Naik, 2020; Rutkowska et al., 2024).

Muscle activation magnitude was quantified using RMS values computed over time windows corresponding to each movement step. The resulting RMS values were normalized to the peak RMS amplitude observed across all trials of the entire session for each participant and muscle, yielding a single fixed reference value per muscle per participant. Because this fixed denominator is applied equally to Step 1 and Step 15, the pre-to-post comparison is not affected by changes in movement amplitude between steps. Pre-analysis quality control included inspection of all EMG signals for artifacts; two participants were excluded due to technical recording failure, and no further exclusions were required. This within-subject normalization approach is well-established in the biomechanical EMG literature (Hermens et al., 2000; De Luca, 1997).

To assess changes in activation variability across intervention steps, the coefficient of variation (CV) of normalized RMS-EMG amplitudes was also computed using Equation 3:


$$\begin{array}{l} CV=\frac{\sigma }{\mu }\times 100, \end{array}$$ (3)

where σ is the standard deviation and μ is the mean RMS amplitude across repetitions, following standard statistical definitions.

### 2.5 Computation of movement smoothness

Movement smoothness during voluntary rotation was quantified using a normalized jerk cost derived from the three-dimensional wrist trajectory. Jerk was defined as the third temporal derivative of position.

The mean squared jerk was computed using Equation 4:


$$\begin{array}{l} J=\frac{1}{T}\int_{0}^{T}||\frac{d^{3}\text{x}\left(t\right)}{dt^{3}}|{|}^{2}dt, \end{array}$$ (4)

where **x**(*t*) is the wrist trajectory and *T* is movement duration. This formulation represents the mean squared jerk over time and corresponds to a time-normalized version of the classical jerk cost proposed by Flash and Hogan (1985).

To enable comparison across trials with different durations and amplitudes, the jerk cost was further normalized as shown in Equation 5:


$$\begin{array}{l} J_{norm}=\frac{J\cdot T^{5}}{A^{2}}, \end{array}$$ (5)

where *A* is the Euclidean displacement between initial and final wrist positions. This normalization yields a dimensionless smoothness index that reduces the influence of both movement duration and amplitude, consistent with previously reported formulations of normalized jerk (Flash and Hogan, 1985; Kitazawa et al., 1993).

A detailed derivation of the normalization procedure is provided in Appendix A.

### 2.6 Ventilatory measurements and movement–ventilation index

Respiratory rate and end-tidal carbon dioxide (ETCO_2_) were continuously monitored during movement using a portable mainstream capnography module (EMMA^TM^, Masimo Corp., Irvine, CA, USA) connected to a breathing mask. ETCO_2_ (mmHg) and breathing rate (breaths/min) were recorded at Step 1 and Step 15 of the protocol.

To relate ventilatory demand to movement amplitude, a ventilatory–movement efficiency index (VMEI) was calculated as shown in Equation 6:


$$\begin{array}{l} VMEI=\frac{ETCO_{2}}{ROM_{\text{Body}}}, \end{array}$$ (6)

where ETCO_2_ denotes end-tidal carbon dioxide (mmHg) and ROM_Body_ denotes the global body range of motion (degrees) defined as the angular excursion of the sacrum-to-wrist vector in the horizontal plane (see below). This index was used as a normalized measure of ventilatory load relative to movement amplitude (Rassler and Raabe, 2003; Bernasconi et al., 1995).

#### 2.6.1 Body Range of motion estimation for VMEI

To compute the movement amplitude used in the VMEI, a segment-based angular measure was defined using the orientation of the vector connecting the sacrum (reference point) to the wrist (end-effector), projected onto the horizontal (X–Y) plane. For each time frame, the instantaneous angle was calculated relative to the global horizontal axis using the two-argument arctangent function (Equation 7):


$$\begin{array}{l} \phi \left(t\right)=\text{atan}2\left(y\left(t\right),x\left(t\right)\right) \end{array}$$ (7)

where *x*(*t*) and *y*(*t*) represent the horizontal components of the sacrum-to-wrist vector. The angle signal was subsequently unwrapped to ensure temporal continuity.

Movement amplitude (ROM_Body_) was then defined as the difference between the mean values of the dominant high peaks and low valleys of the angular trajectory, where peaks and valleys correspond to the dominant local maxima and minima of the angular trajectory identified using a prominence-based criterion described in Equation 2.

This procedure provides a robust estimate of the effective excursion of the distal segment relative to the body while reducing sensitivity to noise and transient fluctuations. This definition differs from the joint-specific ROM measures described above: whereas those measures capture individual segment excursions relative to the pelvis, ROM_Body_ reflects the total amplitude of the distal limb arc through space and is therefore more appropriate as the denominator of the VMEI.

### 2.7 Statistical analysis

All analyses were performed in MATLAB (MathWorks Inc., Natick, MA, USA). Linear mixed-effects models (LMM) were used to account for repeated measurements and between-subject variability. Fixed effects included Time (pre vs. post), Group (intervention vs. control), and their interaction (Time × Group), with Participant included as a random effect.

Separate models were fitted for ROM at each marker, normalized EMG activity for each muscle, forearm angle, normalized jerk cost, and the ventilatory outcomes (ETCO_2_, ROM_Body_, and VMEI). The primary effect of interest was the Time × Group interaction, indicating whether pre–post changes differed between groups; pairwise comparisons were derived from model contrasts. Two linear mixed-effects models were fitted for each outcome. The first evaluated pre–post change separately within each group, with Time as a fixed effect and participant as a random intercept; this model underlies the within-group *p*-values reported in Section 3. The second included Time, Group, and their interaction (Time × Group), with participant as a random intercept and intervention/Step 1 as reference levels, to test directly whether pre–post change differed between groups. Intervention-specific effects are interpreted as confirmed using the Benjamini-Hochberg false discovery rate (FDR) procedure (Benjamini and Hochberg, 1995; Benjamini and Yekutieli, 2001). Where the FDR-corrected Time × Group interaction is statistically significant, effects are reported as confirmed; otherwise, within-group changes are reported descriptively as a consistent pattern and interpreted in light of the power limitation described above.

To account for multiple comparisons across muscles and outcome measures, *p*-values were adjusted using the Benjamini-Hochberg FDR procedure, as described above. Statistical significance was set at adjusted *p* < 0.05. Linear mixed-effects models were fitted in MATLAB (fitlme) using the default estimation procedure, restricted maximum likelihood (REML). The FitMethod='ML' option was not specified. For each outcome family, the analyzed sample consisted of participants with both Step 1 and Step 15 measurements available for that outcome: the ROM, EMG, normalized jerk, and forearm-angle analyses included 44 intervention and 12 control participants, whereas the ventilatory analyses (ETCO_2_, ROM_Body_, VMEI) included 12 participants per group (Section 2.6). No participant included in a given analysis had missing pre- or post-intervention data for that outcome, so all reported models were fitted on complete cases without imputation. The fixed-effect estimates, standard errors, test statistics, degrees of freedom, 95% confidence intervals, raw *p*-values, and FDR-adjusted *p*-values for all 19 outcomes are reported in Table 2.

**Table 2:** Time × Group interaction estimates for all primary outcomes.

Outcome	*N* (Int/Ctrl)	*B*	SE	*t*	DF	95% CI	Raw *p*	FDR-adjusted *p*	Sig.
ROM family (FDR within 12 tests)									
ROM – Head	44/12	−2.973	1.351	−2.201	108	[−5.652, −0.295]	0.030	0.054	No
ROM – Shoulder	44/12	−3.376	1.280	−2.638	108	[−5.913, −0.839]	0.010	0.023	Yes
ROM – Elbow	44/12	−5.388	2.470	−2.181	108	[−10.283, −0.492]	0.031	0.054	No
ROM – Wrist	44/12	−6.981	4.036	−1.730	108	[−14.981, 1.018]	0.087	0.130	No
EMG family (FDR within 27 tests)									
EMG – Sterno-R	44/12	−0.306	0.087	−3.517	108	[−0.479, −0.134]	<0.001	0.006	Yes
EMG – Sterno-L	44/12	−0.119	0.101	−1.178	108	[−0.318, 0.081]	0.241	0.591	No
EMG – Obli-R	44/12	−0.042	0.071	−0.592	108	[−0.182, 0.098]	0.554	0.777	No
EMG – Obli-L	44/12	−0.027	0.097	−0.278	108	[−0.220, 0.166]	0.782	0.878	No
EMG – Erec-Low-R	44/12	−0.021	0.078	−0.269	108	[−0.175, 0.133]	0.789	0.878	No
EMG – Erec-Low-L	44/12	−0.102	0.076	−1.342	108	[−0.253, 0.049]	0.185	0.500	No
EMG – Erec-High-R	44/12	−0.033	0.076	−0.434	108	[−0.183, 0.118]	0.667	0.858	No
EMG – Erec-High-L	44/12	−0.041	0.066	−0.621	108	[−0.171, 0.089]	0.537	0.777	No
EMG – Sol-R	44/12	−0.023	0.098	−0.235	108	[−0.218, 0.172]	0.816	0.878	No
Jerk cost / forearm angle family (FDR within six tests)									
Normalized Jerk	44/12	0.972	1.006	0.966	108	[−1.022, 2.966]	0.336	0.336	No
Forearm Angle	44/12	−11.544	4.408	−2.619	108	[−20.281, −2.808]	0.010	0.030	Yes
Ventilatory family (FDR within nine tests)									
ROM Body	12/12	−7.729	3.246	−2.380	44	[−14.272, −1.187]	0.022	0.065	No
ETCO_2_	12/12	−0.167	1.042	−0.160	44	[−2.267, 1.933]	0.874	0.874	No
VMEI	12/12	0.353	0.180	1.961	44	[−0.010, 0.716]	0.056	0.126	No

Values are fixed-effect estimates for the Time × Group interaction. *B* = regression coefficient; SE, standard error; *t*, test statistic; DF, degrees of freedom; CI, 95% confidence interval; Raw *p*, unadjusted *p*-value; FDR-adjusted *p*, Benjamini–Hochberg adjusted *p*-value. FDR correction was applied separately within each outcome family across the non-intercept fixed effects (Time, Group, and Time × Group). The intervention group and Step 1 were specified as the reference levels. A significant interaction indicates that the pre–post change differed between the intervention and control groups. “Yes” denotes statistical significance after FDR correction.

We note that the power of the Time × Group interaction test was substantially limited by the small control group sample (*n* = 12), as confirmed by the post-hoc sensitivity analysis in Section 4.6; the absence of significant interactions for most outcomes should be interpreted in light of this power limitation.

## 3 Results

### 3.1 Range of motion

The range of motion (ROM) results are shown in Figure 5. In the intervention group, ROM increased significantly from Step 1 to Step 15 for head rotation (*p* < 0.01), shoulder (*p* < 0.01), elbow (*p* < 0.01), and wrist movement (*p* < 0.01). In the control group, ROM did not change significantly for head, shoulder, or wrist (all *p*>0.05); however, elbow ROM decreased significantly from Step 1 to Step 15 (*p* < 0.001, *d*_*z*_ = −1.77), corresponding to a 21.8% decrease, a finding that survived FDR correction. ROM increases in the intervention group were 68.3% for head rotation, 32.1% for shoulder, 48.9% for elbow and 33.5% for wrist movement. Pairwise differences in the magnitude of ROM change across the four markers were not statistically significant. Group-level Step 1, Step 15, and change-score descriptive statistics for this outcome are provided in Appendix Table B1.

**Figure 5:**
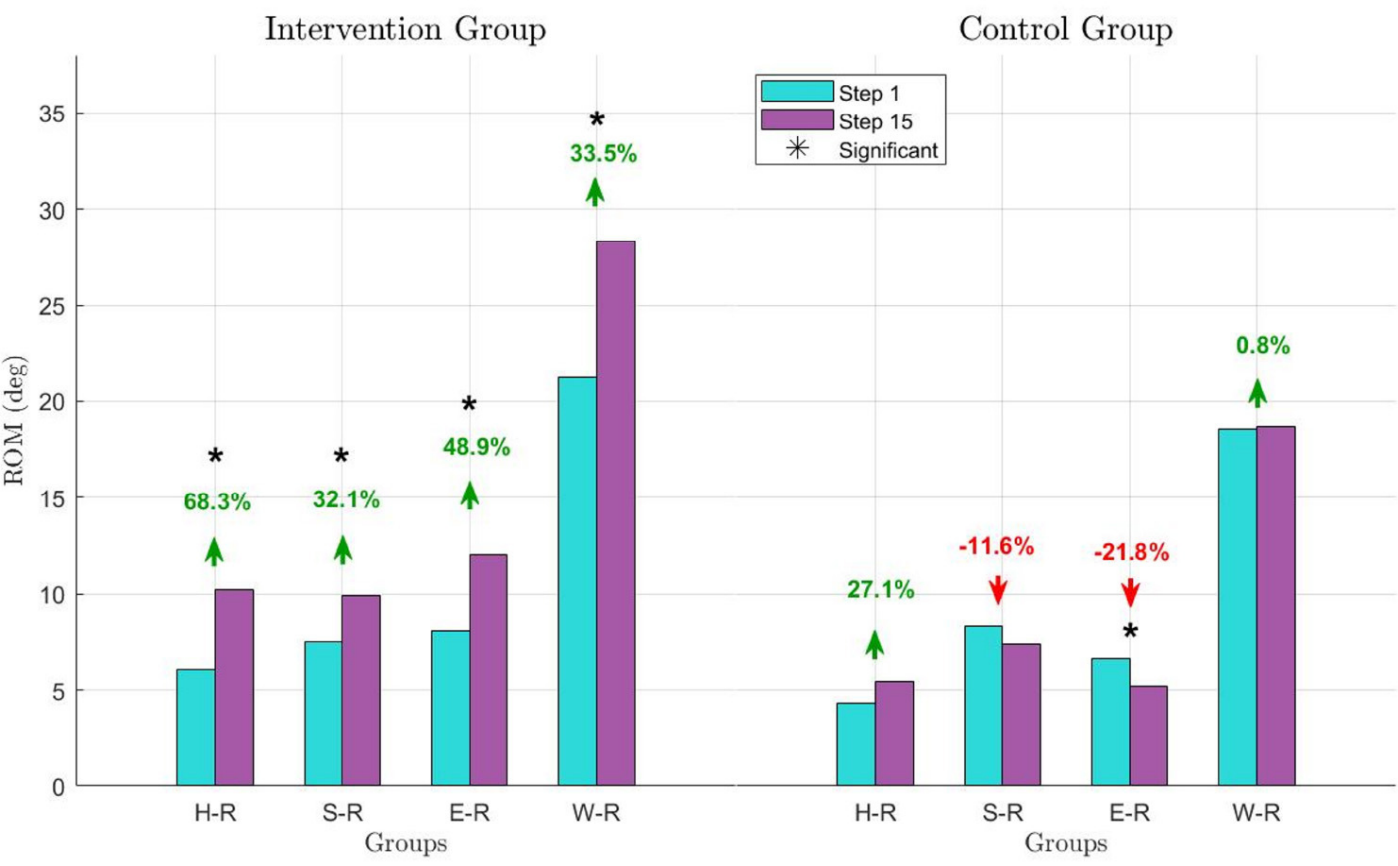
Range of motion (ROM) at Step 1 (pre) and Step 15 (post) for head rotation (H-R), shoulder (S-R), elbow (E-R), and wrist movement (W-R) in the intervention (left) and control (right) groups. Vertical arrows indicate the relative percentage change from Step 1 to Step 15. The asterisk (*) indicates a significant pre–post difference within the respective group (*p* < 0.05).

### 3.2 Electromyographic activity

Changes in EMG activity are summarized in Figure 6. The intervention group showed significant increases from Step 1 to Step 15 in Sterno-R, Sterno-L, Erec-Low-R, Erec-Low-L, and Erec-High-R. All significantly changed muscles showed increases exceeding 20%, with Sterno-R demonstrating the largest change (84%). In the control group, modest increases were observed in some muscles (Erec-Low-R, Erec-High-R, Obli-L), but none reached statistical significance. Group-level Step 1, Step 15, and change-score descriptive statistics for this outcome are provided in Appendix Table B2).

**Figure 6:**
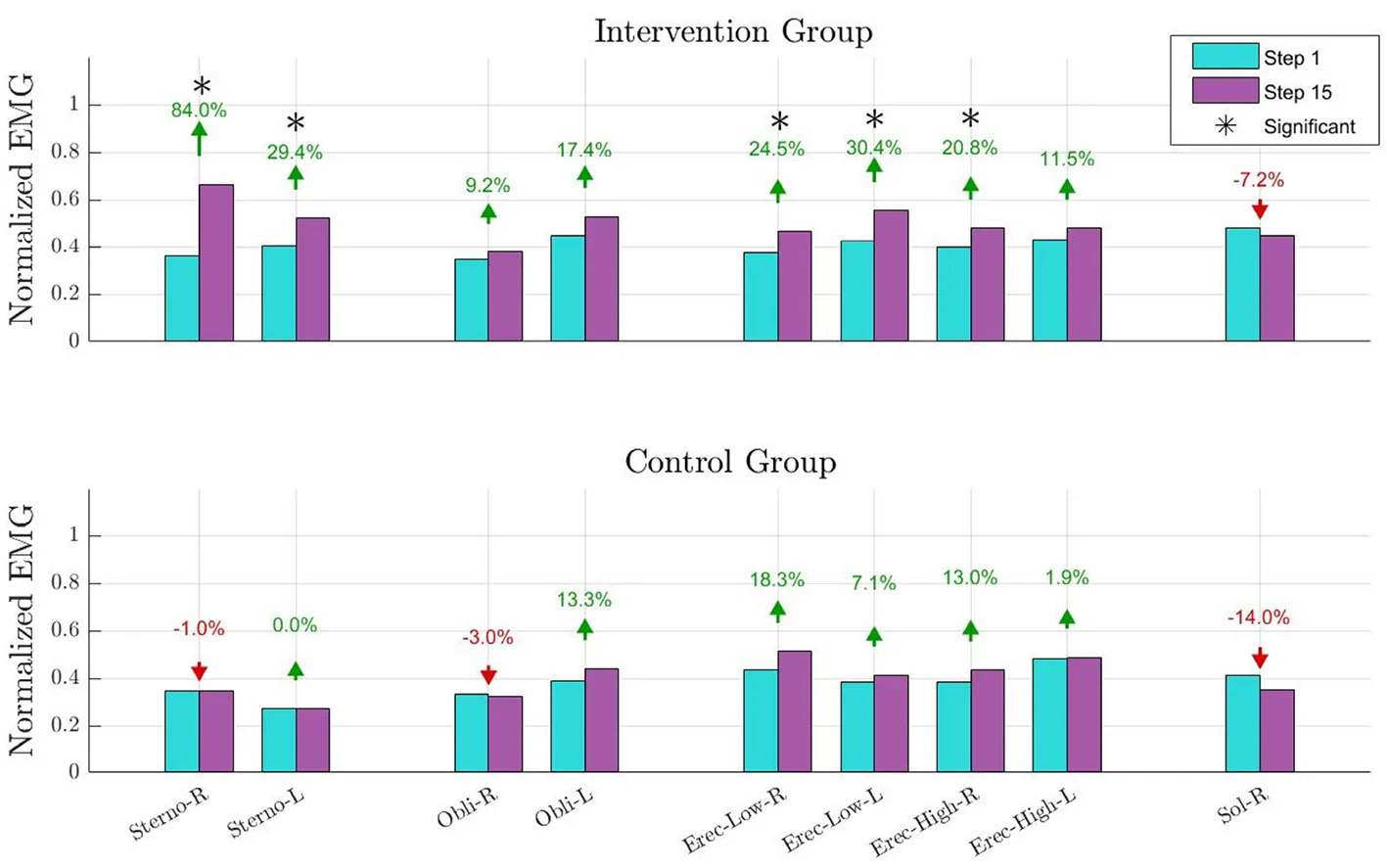
Changes in normalized EMG activity between Step 1 (pre) and Step 15 (post) for both Intervention (top) and Control (bottom) groups. Significant pre–post differences (*, *p* < 0.05) are indicated by asterisks.

As a sensitivity analysis, the EMG Time × Group models were repeated using log-transformed raw RMS amplitudes without normalization. The pattern of inference was unchanged: Sterno-R remained the only muscle showing a statistically significant Time × Group interaction after FDR correction (*B* = −0.913, 95% CI [−1.424, −0.402], raw *p* < 0.001, FDR-adjusted *p* = 0.008), whereas no other muscle showed a significant interaction after correction (Appendix Table B5).

### 3.3 Movement quality

Forearm angle and normalized jerk cost (intervention: *n* = 44; control: *n* = 12) were used to characterize movement quality in terms of segmental contribution and trajectory smoothness, respectively.

Figure 7 shows that in the intervention group, mean forearm angle increased significantly from Step 1 to Step 15 (*p* = 0.005), corresponding to a 6.3% increase. In contrast, the control group exhibited a significant decrease of 4.9% over the same interval (*p* = 0.03). Group-level Step 1, Step 15, and change-score descriptive statistics for forearm angle are provided in Appendix Table B3.

**Figure 7:**
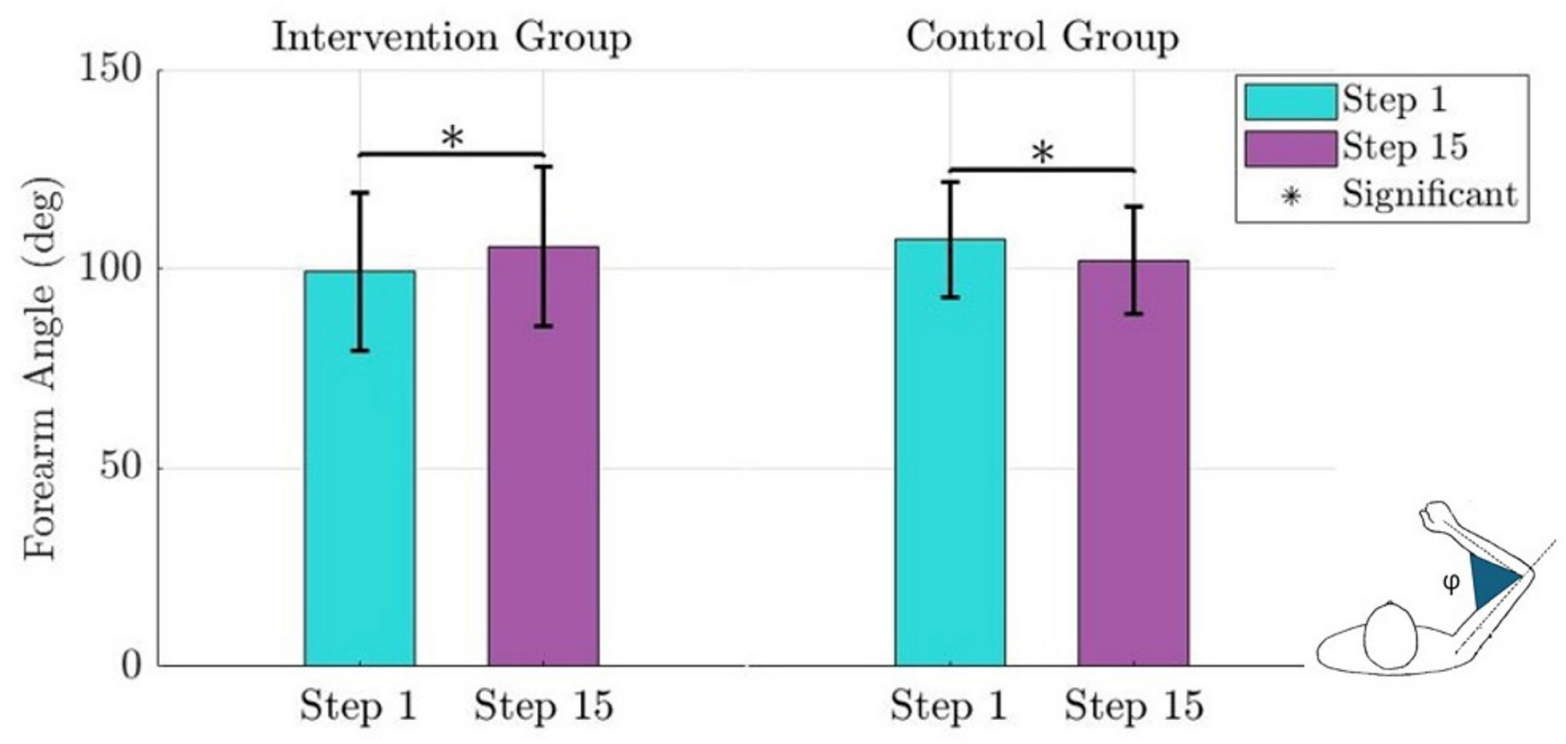
Mean ± SD values of forearm angle at Step 1 and Step 15 for the intervention and control groups. The left panel represents the intervention group and the right panel the control group. A significant pre–post change was observed within each group (*p* < 0.05), as indicated by the asterisk.

Normalized jerk cost, shown in Figure 8, increased significantly in both groups (intervention: *p* = 0.02; control: *p* = 0.01, corresponding to increases of 54.1 and 224.6% in the intervention and control groups, respectively), indicating reduced movement smoothness over repeated execution. Group-level Step 1, Step 15, and change-score descriptive statistics for normalized jerk cost are provided in Appendix Table B4. The increase in jerk cost was numerically larger in the control group than in the intervention group, indicating a greater loss of smoothness with repeated execution in the absence of the intervention.

**Figure 8:**
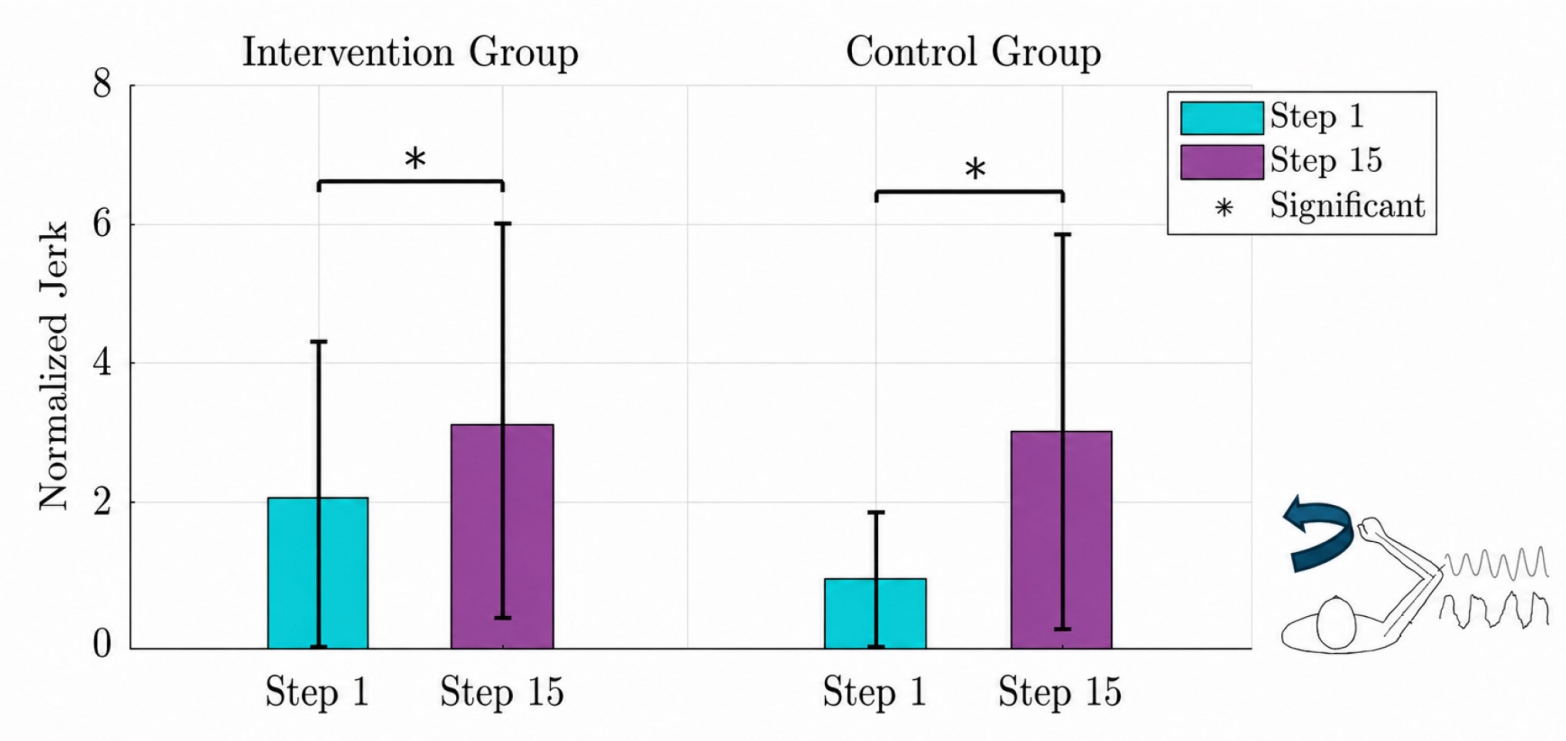
Mean ± SD values of normalized jerk at Step 1 and Step 15 for the intervention and control groups. The left panel corresponds to the intervention group, while the right panel represents the control group. Significant differences between steps within each group are marked with an asterisk (*p* < 0.05).

### 3.4 Movement efficiency

Movement efficiency was assessed using the ventilatory–movement efficiency index (VMEI; see the Ventilatory Measurements subsection in Methods).

Individual VMEI values for both groups are illustrated in Figure 9. In the intervention group, ROM_Body_ increased significantly from Step 1 to Step 15 (26.3%), while VMEI decreased significantly (20%), indicating that a larger movement amplitude was achieved with comparable ventilatory demand, consistent with a more favorable ventilatory cost relative to movement amplitude (see Section 4.4 for interpretation). ETCO_2_ did not change significantly in either group. In the control group, no significant changes were observed in ROM_Body_, ETCO_2_, or VMEI.

**Figure 9:**
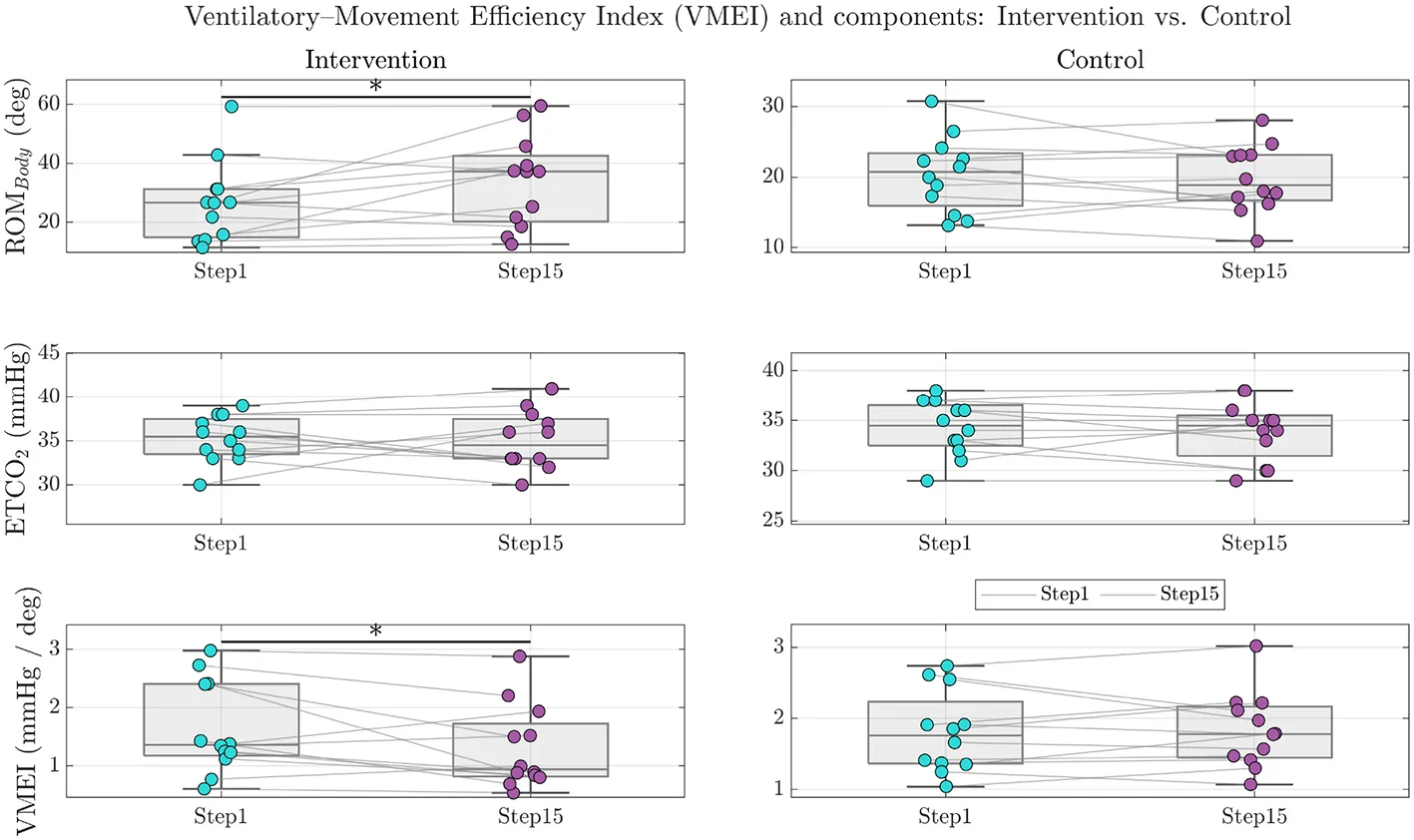
Ventilatory–movement efficiency index (VMEI) and its components (ROM_Body_, ETCO_2_) at Step 1 and Step 15, for the intervention (left) and control (right) groups. Each point represents an individual participant; thin gray lines connect each participant's Step 1 and Step 15 values, and box plots show the median, interquartile range, and full range across participants at each time point. Note that *y*-axis ranges differ between the intervention and control panels within a row, reflecting differences in movement amplitude and ventilatory values between groups. Asterisks (*) denote a significant pre–post change at the group level (*p* < 0.05).

## 4 Discussion

This study investigated whether a single Feldenkrais Awareness Through Movement session produces measurable changes in voluntary movement organization using objective laboratory measurements. While previous research has documented clinical benefits of Feldenkrais-based practice (Berland et al., 2022; Hillier and Worley, 2015), the neuromechanical mechanisms underlying reported improvements in ROM and functional movement have remained largely uncharacterized. The present findings reveal coordinated changes across kinematic, neuromuscular, and ventilatory domains that are specific to the intervention group, advancing the mechanistic understanding of Feldenkrais-based movement practice beyond what clinical and self-report outcomes can offer.

### 4.1 Range of motion and intersegmental coordination

One central question motivating this study was whether increases in voluntary ROM following Feldenkrais-based practice reflect changes in passive joint mechanics or, instead, reflect a reorganization of how motion is distributed across body segments. The ROM increases observed in the intervention group across all measured upper-body segments (Figure 5) were obtained in the absence of any external stretching, force application, or explicit ROM instructions, making a purely mechanical explanation unlikely. The control group, who performed an equivalent volume of repeated rotations, showed no significant ROM changes at head, shoulder, or wrist markers. Notably, elbow ROM decreased significantly in the control group (*p* < 0.001, *d*_*z*_ = −1.77), opposite in direction to the significant elbow ROM increase observed in the intervention group, suggesting that repeated stereotyped movement without exploratory guidance may reduce rather than maintain elbow excursion over a single session. The Time × Group interaction was statistically significant for shoulder ROM after FDR correction (*p* = 0.023; Table 2), whereas the corresponding interactions for head, elbow, and wrist ROM did not reach significance after FDR correction, reflecting the limited power of the interaction test given the small control group. The absence of a control-group ROM increase therefore provides support against a simple repetition-based explanation and, taken together with the consistent pattern of intervention-group gains across all four markers, The absence of a control-group ROM increase therefore provides support against a simple repetition-based explanation and, taken together with the consistent pattern of intervention-group gains across all four markers, is consistent with the possibility that the ATM content contributed to the observed kinematic changes, with the shoulder ROM interaction providing direct statistical support for an intervention-specific effect. Rather, the pattern is consistent with a coordination-based mechanism in which ATM practice progressively releases habitual constraints on segmental motion, in line with Bernstein's framework of degrees-of-freedom regulation during skill acquisition (Bernstein, 1967). Previous systematic work has similarly attributed ROM and functional gains following Feldenkrais interventions to changes in movement strategy and coordination rather than to structural tissue change (Berland et al., 2022; Hillier and Worley, 2015), and the present findings provide direct kinematic evidence in support of this interpretation.

The group-specific change in forearm angle (Figure 7) adds a further dimension to this picture. The intervention group showed a significant increase from Step 1 to Step 15, confirmed by a significant Time × Group interaction after FDR correction (*p* = 0.030; Table 2). Forearm angle reflects the inter-segment configuration at the elbow joint, and an increase during the rotation task is consistent with the Feldenkrais hypothesis that exploratory practice promotes more active multi-joint participation. The present findings support the effectiveness of the intervention in producing group-specific changes in upper-limb configuration, a direction of change that is inherently positive in the context of movement rehabilitation, where increased ROM and broader segmental engagement are therapeutic goals (Berland et al., 2022; Ullmann et al., 2010). However, we note that whether this reflects improved intersegmental coordination specifically requires further investigation with direct coordination measures.

### 4.2 Neuromuscular activation and coordination

A core premise of Feldenkrais-based practice is that functional movement improvements arise from changes in how the nervous system organizes muscle activation, rather than from increases in maximal force capacity (Newell, 1990; Mattes, 2016). The present EMG findings bear directly on this premise. Voluntary trunk rotation involves coordinated activation of neck, trunk, and postural muscles, and the pattern of muscle recruitment reflects both the intended movement task and the strategy by which degrees of freedom are regulated across segments (Bernstein, 1967; Winter, 2009).

Selective and significant increases in muscle activation were observed for Sterno-R, Sterno-L, Erec-Low-R, Erec-Low-L, and Erec-High-R in the intervention group, with Sterno-R also showing a significant Time × Group interaction, providing direct evidence of a group-specific neuromuscular response. No significant changes were detected in the control group. The muscles that changed are functionally relevant: the sternocleidomastoid contributes to head and neck rotation, while the erector spinae bilaterally supports and organizes spinal extension and axial rotation (Winter, 2009). That activation in these muscles increased alongside ROM suggests a more engaged and distributed recruitment strategy rather than a passive release of restrictions. This is consistent with the view that greater voluntary excursion emerged from more complete use of available degrees of freedom (e.g., through improved intersegmental coordination), rather than from a reduction in passive joint stiffness or increased maximal muscular force. The increased muscle activation also aligns with the greater movement amplitude associated with the larger ROM excursion.

The observed increases in EMG variability within the intervention group are consistent with the concept of adaptive motor exploration described by Stergiou et al. (2006), under which increased motor variability during motor learning reflects the motor system searching for more efficient coordination solutions within existing biomechanical constraints (Appendix Table B7). This theoretical perspective aligns well with the self-regulated, non-prescriptive nature of ATM practice and warrants investigation with direct measures of neural activity and learning retention in future work.

### 4.3 Movement quality: smoothness and segmental contribution

Movement quality encompasses not only amplitude but also the manner in which trajectories are executed. Trajectory smoothness, as indexed by normalized jerk cost, provides a kinematic correlate of movement coordination quality that is independent of speed and amplitude, and has been used to characterize motor learning and neurological rehabilitation outcomes (Flash and Hogan, 1985; Kitazawa et al., 1993).

Both groups showed a significant increase in jerk cost over the session (Figure 8), indicating that neither group improved trajectory smoothness within a single session. The increase in normalized jerk cost differed substantially in magnitude: 54.1% in the intervention group vs. 224.6% in the control group. We note that the corresponding Time × Group interaction was not statistically significant after FDR correction (*p* = 0.336; Table 2), reflecting the limited power of the interaction test given the small control group. This difference in percentage change therefore does not provide formally confirmed evidence of a differential or protective effect of ATM practice on trajectory smoothness. Nonetheless, both groups started from a comparable baseline, making a ceiling or floor effect unlikely as an explanation for the magnitude difference. We offer the following as a plausible, but statistically unconfirmed, interpretation: the exploratory coordination demands of ATM practice may attenuate the degradation in smoothness that typically accompanies repeated stereotyped movement execution, a hypothesis warranting investigation with larger and more balanced samples.

The forearm angle and jerk cost findings together suggest that Feldenkrais practice affects movement quality through two partially independent pathways: by expanding the spatial distribution of movement across segments, and by possibly attenuating the decline in trajectory smoothness over repeated execution, a hypothesis that requires confirmation in adequately powered studies.

### 4.4 Ventilatory-movement index

A reduction in VMEI, as observed here only in the intervention group (−20%, Figure 9), largely reflects the significant increase in ROM_Body_, given that ETCO_2_ did not change significantly in either group. We note, however, that ETCO_2_ is a proxy for ventilatory drive rather than a direct measure of metabolic cost or energy expenditure; confirming a genuine improvement in metabolic efficiency would require direct gas exchange analysis (VO_2_) or heart-rate-based measures, neither of which was collected here. In the control group, no significant changes in ROM_Body_, VMEI, or ETCO_2_ were observed. The Time × Group interactions for ROM_Body_ (*p* = 0.065) and VMEI (*p* = 0.126) did not reach significance after FDR correction, again reflecting the limited power of the interaction test for these outcomes. The consistent pattern of intervention-group improvement without corresponding control-group change is therefore discussed cautiously as a descriptive pattern rather than as evidence of a formally confirmed differential effect. The present results extend this work by showing that the ventilatory cost per unit of movement amplitude can be reduced through coordinated intersegmental practice; we frame this as a change in movement–ventilation coupling rather than as direct evidence of improved metabolic efficiency, which future work using gas exchange or heart-rate telemetry would need to confirm.

### 4.5 Broader implications

Considered together, these findings present a coherent picture of how a single Feldenkrais ATM session modifies movement organization across multiple levels of analysis. Increased ROM and forearm angle reflect changes in the spatial distribution of movement; selective EMG increases and structured variability (Appendix Table B7) reflect changes in neuromuscular coordination strategy; partially preserved smoothness reflects changes in trajectory quality; and reduced VMEI reflects changes in movement economy. That these changes occurred together, and specifically in the intervention group, supports the interpretation that ATM practice induced a reorganization of motor coordination rather than a non-specific effect of repeated movement.

From a rehabilitation perspective, these results suggest that coordination-based motor training can alter movement organization even within a single session, without external force, stretching, or performance feedback. This has practical relevance for the design of movement rehabilitation programs, particularly for populations in which conventional strength- or flexibility-based approaches are contraindicated or insufficient. Furthermore, the multidimensional assessment framework employed here, combining kinematics, EMG, and ventilatory measures, offers a useful model for evaluating coordination-based therapeutic interventions in future research. These principles may also inform the design of assistive and rehabilitation systems such as wearable exoskeleton controllers, biofeedback-guided neurorehabilitation platforms, or sensory augmentation interfaces that aim to support adaptive movement coordination and sensory awareness rather than simply augmenting force or restricting compensatory motion. These represent potential future directions motivated by the concepts and hypotheses developed in this study, requiring clinical and technological validation.

### 4.6 Limitations and future directions

Several limitations of the present study should be acknowledged. First, the findings reflect acute adaptations following a single ATM session; the control group confirms that these changes are not attributable to repetition alone, but it remains unclear whether effects are maintained or further amplified with repeated practice over time. Second, all participants were young healthy adults, which limits the generalizability of the findings to clinical or older populations in whom Feldenkrais-based practice is more commonly applied. Third, the control condition was matched to the intervention in movement dose (duration and repetition count), rest intervals, practitioner supervision, and general movement-performance instructions, as described in the Methods. It was not designed to reproduce the specific content of the ATM lessons, including their progressive movement variety, attentional guidance toward specific body segments, segmental differentiation instructions, and novelty across steps. This is consistent with the study's goal of showing that the Feldenkrais Method has effects beyond those of physical repetition alone. Future investigations could employ an attention-matched active control to further characterize which specific components of ATM practice drive the observed effects. Cohen's *d*_*z*_ and 95% confidence intervals for all within-group pre–post comparisons are provided in Appendix Table B6. The one exception–a significant elbow ROM decrease (*d*_*z*_ = −1.77, 95% CI [−2.674, −0.829])–was opposite in direction to the intervention-group ROM increase, but this contrast should be interpreted cautiously and does not by itself establish an intervention-specific effect. For the control group (*n* = 12), most ROM and EMG comparisons were non-significant, meaning that these results do not rule out small effects of repetition and should therefore be interpreted cautiously when considering the magnitude of change observed in the intervention group.

The limited power of the Time × Group interaction test for most outcomes (5%–21% for ROM and EMG) reflects the design constraint of the control group, which was sized to provide a comparison for repetition-based explanations rather than to power a formal interaction comparison. Future studies with larger and more balanced group sizes are needed to draw firm conclusions about intervention specificity for these outcomes.

The within-group effect sizes in Appendix Table B6 further contextualize these power limitations: the intervention group showed medium to large effects for the significant outcomes (e.g., Sterno-R: *d*_*z*_ = 1.024, Head ROM: *d*_*z*_ = 0.930, Shoulder ROM: *d*_*z*_ = 0.585), while the control group showed near-zero or negative values for the same outcomes, providing additional support for the intervention-specific nature of the observed changes despite the limited power of the formal interaction tests.

A further limitation concerns the VMEI metric itself. End-tidal CO_2_ is a proxy for ventilatory drive rather than a direct measure of metabolic cost or energy expenditure, and the VMEI is accordingly best interpreted as an index of ventilatory load relative to movement amplitude rather than of true metabolic efficiency. Confirming whether the observed reduction in VMEI corresponds to a genuine improvement in metabolic economy would require direct gas exchange analysis (VO_2_) or heart-rate-based measures, neither of which was included in the present protocol. Future investigations should examine longer-term adaptations across multiple sessions, incorporate additional muscle groups and neural activity measures to further characterize sensorimotor reorganization, and extend the framework to clinical populations in which exploratory movement practice may have direct therapeutic relevance.

## 5 Conclusion

This study provides objective, laboratory-based evidence that a single Feldenkrais Awareness Through Movement session produces coordinated changes in voluntary movement organization across kinematic, neuromuscular, and ventilatory domains in healthy adults. ROM increased significantly in all measured upper-body segments of the intervention group, while the control group showed no significant ROM increases at any marker. The one significant control-group change, a decrease in elbow ROM, was opposite in direction to the intervention-group gains. The Time × Group interaction was statistically significant for shoulder ROM after FDR correction (*p* = 0.023), providing direct evidence of a differential pre–post change between groups. In contrast, the interactions for head, elbow, and wrist ROM did not reach significance after FDR correction and are therefore interpreted as consistent patterns rather than formally confirmed intervention-specific effects. Selective increases in muscle activation in functionally relevant trunk and neck muscles, alongside group-specific forearm angle changes, indicate that ROM improvements reflect a reorganization of intersegmental coordination rather than passive mechanical release. Although jerk cost increased in both groups, the numerically smaller increase in the intervention group is interpreted descriptively as consistent with a protective effect of ATM practice on trajectory smoothness; however, the Time × Group interaction was not statistically significant, thus it should be regarded as a hypothesis-generating observation rather than a confirmed finding. The reduction in VMEI in the intervention group, in the absence of any change in ETCO_2_, indicates that greater movement amplitude was achieved with comparable ventilatory demand, consistent with a change in movement–ventilation coupling rather than a reduction in metabolic cost per se. Together, these findings support a neuromechanical account of Feldenkrais-based movement improvement and demonstrate that objective kinematic, EMG, and ventilatory assessment can capture meaningful changes in movement coordination that clinical scales alone cannot. By establishing that exploratory coordination-based practice can modify movement organization in a single session, this work opens pathways for applying Feldenkrais principles in rehabilitation and in the design of assistive technologies that prioritize adaptive, sensory-informed movement over purely biomechanical correction.

## Data Availability

The raw data supporting the conclusions of this article will be made available by the authors, without undue reservation.
