# Supplementary material for: Acute neuromuscular and kinematic responses to a single Feldenkrais awareness through movement session
Source: Front Hum Neurosci. 2026 Oct 7;20:1924684. doi: 10.3389/fnhum.2026.1924684 (PMC13683800; doi:10.3389/fnhum.2026.1924684)
Supplement: Supplementary file 1 [file Supplementary_file_1.pdf]

APPENDIX A. DERIVATION AND NORMALIZATION OF THE JERK METRIC

A.1 Classical Jerk Cost

The classical smoothness formulation, introduced by Flash and Hogan, is based on the integral of squared jerk:

$$J_c = \int_0^T \left\| \frac{d^3x(t)}{dt^3} \right\|^2 dt \tag{8}$$

This quantity represents a cumulative jerk cost over the duration of the movement.

A.2 Mean Squared Jerk Formulation

In this study, the jerk cost is reformulated as a mean quantity over time. The mean squared jerk formulation used in the main text (Eq. (5)) is reproduced here for completeness:

$$J = \frac{1}{T} \int_0^T \left\| \frac{d^3x(t)}{dt^3} \right\|^2 dt \tag{9}$$

This expression is obtained by defining the mean squared jerk as:

$$J = \frac{J_c}{T} \tag{10}$$

This step represents a normalization with respect to movement duration, converting the measure from a cumulative quantity into an average one.

A.3 Dimensional Analysis

To construct a scale-independent measure, the physical dimensions of the jerk term are examined.

Position has dimension:

$$[x] = L$$

Time has dimension:

$$[t] = T$$

The third derivative of position (jerk) has dimension:

$$\frac{d^3x}{dt^3} \sim \frac{L}{T^3}$$

Squaring yields:

$$\left(\frac{d^3x}{dt^3}\right)^2 \sim \frac{L^2}{T^6}$$

Integration over time gives:

$$J_c \sim \frac{L^2}{T^5}$$

Including the factor $1/T$, the mean squared jerk becomes:

$$J \sim \frac{L^2}{T^6}$$

A.4 Normalization

The quantity J still depends on both time and spatial scale. To reduce these dependencies, a normalization factor is introduced:

$$\frac{T^5}{A^2}$$

where A is defined as the Euclidean displacement between the initial and final positions:

$$A = \|x(T) - x(0)\| \tag{11}$$

The normalized jerk is then defined as:

$$J_{norm} = J \cdot \frac{T^5}{A^2} \tag{12}$$

Substituting J, we obtain:

$$J_{norm} = \frac{1}{T} \int_0^T \left\| \frac{d^3x(t)}{dt^3} \right\|^2 dt \cdot \frac{T^5}{A^2} \tag{13}$$

This formulation removes the spatial dependence and significantly reduces the temporal dependence, yielding a scale-independent smoothness measure.

A.5 Interpretation

The derivation follows a structured sequence:

- Start from the classical jerk cost formulation
- Reformulate it as a mean quantity using $1/T$
- Perform dimensional analysis explicitly
- Apply normalization using T^5/A^2

A.6 Summary

The derivation above yields a dimensionless, scale-independent jerk-based smoothness metric consistent with the formulations reported in (Flash and Hogan, 1985; Kitazawa et al., 1993). The normalization by T^5/A^2 removes the spatial scale dependence and substantially reduces the temporal dependence, enabling valid comparison of smoothness across trials differing in speed and amplitude.

APPENDIX B. SUPPLEMENTARY CHANGE-SCORE DESCRIPTIVE STATISTICS

To improve transparency regarding individual-level variability underlying the group-level results reported in Sections 3.1–3.3, Tables B1–B4 summarize Step 1, Step 15, and within-participant change-score (Step 15 − Step 1) descriptive statistics (mean ± SD) for the primary outcome families, computed directly from the analyzed participant-level dataset (intervention: $n = 44$; control: $n = 12$). These are group-level mean ± SD summaries calculated from the participant-level dataset and are provided for descriptive transparency only; they do not represent individual-level distributions. Inferential conclusions continue to rest on the linear mixed-effects and FDR-adjusted results reported in Table 2.

Table B5 presents the sensitivity analysis of the EMG Time × Group effects using log-transformed raw RMS amplitudes without normalization. Table B6 presents within-group pre–post effect sizes (Cohen's d_z) with 95% confidence intervals for the primary normalized RMS EMG data. Table B7 provides descriptive coefficients of variation (CV) for normalized RMS EMG values across participants within each group and step.

Table B1. Range of Motion (deg): Step 1, Step 15, and change-score descriptive statistics (mean ± SD) by marker and group.

Marker	Group	n	Step 1	Step 15	Change
Head	Intervention	44	6.06 ± 3.30	10.20 ± 5.54	4.14 ± 4.45
Head	Control	12	4.29 ± 1.76	5.46 ± 3.06	1.17 ± 3.19
Shoulder	Intervention	44	7.52 ± 4.53	9.93 ± 6.50	2.41 ± 4.12
Shoulder	Control	12	8.33 ± 3.02	7.37 ± 2.42	-0.96 ± 3.50
Elbow	Intervention	44	8.08 ± 4.92	12.03 ± 8.32	3.95 ± 8.65
Elbow	Control	12	6.61 ± 1.35	5.17 ± 1.31	-1.44 ± 0.81
Wrist	Intervention	44	21.23 ± 11.39	28.35 ± 16.99	7.12 ± 13.52
Wrist	Control	12	18.58 ± 6.97	18.72 ± 5.77	0.14 ± 8.22

Table B2. Normalized EMG (RMS): Step 1, Step 15, and change-score descriptive statistics (mean ± SD) by muscle and group.

Muscle	Group	n	Step 1	Step 15	Change
Sterno-R	Intervention	44	0.360 ± 0.149	0.663 ± 0.328	0.303 ± 0.296
Sterno-R	Control	12	0.348 ± 0.163	0.345 ± 0.239	-0.003 ± 0.149
Sterno-L	Intervention	44	0.403 ± 0.150	0.522 ± 0.330	0.119 ± 0.329
Sterno-L	Control	12	0.271 ± 0.130	0.271 ± 0.271	0.000 ± 0.251
Obli-R	Intervention	44	0.346 ± 0.115	0.378 ± 0.282	0.032 ± 0.225
Obli-R	Control	12	0.333 ± 0.060	0.323 ± 0.184	-0.010 ± 0.204
Obli-L	Intervention	44	0.451 ± 0.125	0.529 ± 0.365	0.079 ± 0.319
Obli-L	Control	12	0.388 ± 0.142	0.440 ± 0.316	0.052 ± 0.240
Erec-Low-R	Intervention	44	0.366 ± 0.148	0.466 ± 0.306	0.100 ± 0.229
Erec-Low-R	Control	12	0.433 ± 0.151	0.512 ± 0.340	0.079 ± 0.288
Erec-Low-L	Intervention	44	0.425 ± 0.139	0.554 ± 0.296	0.129 ± 0.254
Erec-Low-L	Control	12	0.385 ± 0.103	0.413 ± 0.217	0.028 ± 0.159
Erec-High-R	Intervention	44	0.398 ± 0.153	0.480 ± 0.296	0.083 ± 0.211
Erec-High-R	Control	12	0.384 ± 0.177	0.434 ± 0.336	0.050 ± 0.321
Erec-High-L	Intervention	44	0.431 ± 0.162	0.480 ± 0.253	0.050 ± 0.160
Erec-High-L	Control	12	0.479 ± 0.170	0.488 ± 0.263	0.009 ± 0.326
Sol-R	Intervention	44	0.483 ± 0.217	0.448 ± 0.398	-0.035 ± 0.334
Sol-R	Control	12	0.410 ± 0.181	0.352 ± 0.209	-0.057 ± 0.166

Table B3. Forearm Angle (deg): Step 1, Step 15, and change-score descriptive statistics (mean ± SD) by group.

**Group**	**n**	**Step 1**	**Step 15**	**Change**
Intervention	44	99.340 ± 20.011	105.596 ± 20.253	6.256 ± 14.796
Control	12	107.392 ± 14.565	102.103 ± 13.661	-5.288 ± 8.760

Table B4. Normalized Jerk Cost: Step 1, Step 15, and change-score descriptive statistics (mean ± SD) by group.

**Group**	**n**	**Step 1**	**Step 15**	**Change**
Intervention	44	2.097 ± 2.211	3.231 ± 2.786	1.134 ± 3.218
Control	12	0.938 ± 0.914	3.043 ± 2.801	2.106 ± 2.844

Table B5. Sensitivity analysis of EMG Time × Group interaction estimates using log-transformed raw RMS amplitudes.

Outcome	N (Int/Ctrl)	B	SE	t	DF	95% CI	Raw p	FDR-adjusted p	Sig.
EMG family (FDR within 27 tests)									
EMG – Sterno-R	44/12	−0.913	0.258	−3.543	108	[−1.424, −0.402]	< 0.001	0.008	Yes
EMG – Sterno-L	44/12	−0.551	0.269	−2.049	108	[−1.084, −0.018]	0.043	0.245	No
EMG – Obli-R	44/12	0.102	0.230	0.445	108	[−0.353, 0.558]	0.657	0.998	No
EMG – Obli-L	44/12	−0.007	0.163	−0.042	108	[−0.331, 0.317]	0.966	0.998	No
EMG – Erec-Low-R	44/12	0.018	0.242	0.076	108	[−0.460, 0.497]	0.940	0.998	No
EMG – Erec-Low-L	44/12	−0.103	0.283	−0.364	108	[−0.665, 0.459]	0.717	0.998	No
EMG – Erec-High-R	44/12	−0.383	0.252	−1.517	108	[−0.883, 0.118]	0.132	0.511	No
EMG – Erec-High-L	44/12	−0.148	0.203	−0.728	104	[−0.550, 0.255]	0.468	0.998	No
EMG – Sol-R	44/12	0.030	0.253	0.119	108	[−0.471, 0.531]	0.905	0.998	No

Note. Values are fixed-effect estimates for the Time × Group interaction from linear mixed-effects models using log-transformed raw RMS EMG amplitudes without normalization. B = regression coefficient; SE = standard error; t = test statistic; DF = degrees of freedom; CI = 95% confidence interval; Raw p = unadjusted p-value; FDR-adjusted p = Benjamini–Hochberg adjusted p-value. FDR correction was applied across the 27 non-intercept EMG tests (Time, Group, and Time × Group effects across nine muscles). Intervention and Step 1 were specified as the reference levels. Four non-finite observations were excluded for Erec-High-L, resulting in DF = 104 for this muscle.

Table B6. Within-group pre–post effect sizes (Cohen's d_z) with 95% confidence intervals.

Outcome	Intervention (N)				Control (N)			
	N	d_z	95% CI Lower	95% CI Upper	N	d_z	95% CI Lower	95% CI Upper
Sterno-R	44	1.024	0.654	1.386	12	−0.022	−0.587	0.544
Sterno-L	44	0.361	0.054	0.664	12	0.000	−0.566	0.566
Obli-R	44	0.142	−0.156	0.438	12	−0.049	−0.614	0.518
Obli-L	44	0.247	−0.054	0.546	12	0.215	−0.362	0.783
Erec-Low-R	44	0.436	0.124	0.743	12	0.275	−0.308	0.846
Erec-Low-L	44	0.507	0.190	0.819	12	0.172	−0.402	0.738
Erec-High-R	44	0.393	0.084	0.698	12	0.156	−0.417	0.722
Erec-High-L	44	0.309	0.005	0.610	12	0.027	−0.540	0.592
Sol-R	44	−0.103	−0.399	0.194	12	−0.346	−0.922	0.245
Head ROM	44	0.930	0.571	1.280	12	0.365	−0.228	0.943
Shoulder ROM	44	0.585	0.262	0.902	12	−0.276	−0.847	0.307
Elbow ROM	44	0.457	0.144	0.765	12	−1.766	−2.674	−0.829
Wrist ROM	44	0.527	0.209	0.840	12	0.017	−0.549	0.582
Normalized Jerk	44	0.352	0.046	0.655	12	0.740	0.085	1.371
Forearm Angle	44	0.423	0.112	0.729	12	−0.604	−1.211	0.026
ROMBody	12	0.629	−0.006	1.239	12	−0.195	−0.762	0.381
$ETCO_2$	12	−0.051	−0.616	0.516	12	−0.173	−0.740	0.401
VMI	12	−0.589	−1.194	0.038	12	0.069	−0.499	0.634

Note. Cohen's d_z represents the within-group pre–post effect size calculated from the difference between Step 15 and Step 1. The 95% confidence interval is reported as separate lower and upper limits. These effect sizes represent within-group pre–post changes and do not represent the Time × Group interaction.

Table B7. Coefficient of variation (CV) of normalized RMS EMG by group and step.

Group	Step	CV (%)								
		Sterno-R	Sterno-L	Obli-R	Obli-L	Erec-Low-R	Erec-Low-L	Erec-High-R	Erec-High-L	Sol-R
Intervention	Step 1	41.27	37.28	33.19	27.76	40.34	32.78	38.36	37.71	45.05
	Step 15	49.50	63.27	74.68	68.91	65.70	53.47	61.71	52.56	88.75
Control	Step 1	46.75	48.02	17.97	36.63	34.81	26.73	46.20	35.39	44.10
	Step 15	69.32	99.92	56.79	71.77	66.32	52.50	77.46	53.92	59.21

Note. CV (%) was calculated as SD/mean × 100 using normalized RMS EMG values across participants within each group and step. CV values represent descriptive measures of relative variability and were not subjected to inferential statistical testing.
